# Psychological Distress and Related Variables in Older Adults from Northeast Brazil

**DOI:** 10.3390/bs16081295

**Published:** 2026-07-29

**Authors:** Josevânia da Silva, Barbara Tejo Bezerra Araújo de Souza, Karla Santos Mateus, Emerson Do Bú, Deusivania Vieira da Silva Falcão

**Affiliations:** 1 Department of Psychology, State University of Paraíba, Campina Grande 58429-500, PB, Brazil; josevania.psi@servidor.uepb.edu.br (J.d.S.); karla.mateus@visitante.uepb.edu.br (K.S.M.); 2 Department of Psychology, Federal University of Paraíba, João Pessoa 58051-900, PB, Brazil; psi.barbaratejo@gmail.com; 3 Institute of Social Sciences, University of Lisbon, 1649-004 Lisbon, Portugal; 4 Escola de Artes, Ciências e Humanidades, University of São Paulo, São Paulo 03828-000, SP, Brazil; deusivania@usp.br

**Keywords:** aging, psychological distress, food insecurity, health inequities

## Abstract

Population aging is reshaping public health priorities across Latin America, yet most evidence on late-life psychological distress comes from high-income societies. Brazil’s Northeast combines rapid aging with persistent structural deprivation. The aim of this study was to estimate the prevalence of psychological distress among older adults in this region and to examine its association with food insecurity, sociodemographic position, and health-related behaviors. A convenience sample of 355 adults aged 60 to 94 years was recruited in Paraíba, Piauí, and Bahia. Participants completed a sociodemographic and health questionnaire, the short-form Brazilian Food Insecurity Scale, and the Kessler Psychological Distress Scale (K10). Distress exceeded the validated screening cutoff in 64.5% of the sample, and 40.8% reported food insecurity. Older adults with food insecurity scored higher on the K10 than their food-secure peers (*p* = 0.001, *d* = −0.847), an association that persisted under multivariable adjustment. Lower income, lower schooling, and medication use were also associated with greater distress, and physical activity and life satisfaction with less. Because the design was cross-sectional and the sample non-probabilistic, these associations describe co-occurrence rather than chronicity or causal direction. Even so, the findings support addressing food security alongside mental health care in this population.

## 1. Introduction

Mental health is foundational to well-being in later life. It reflects the interplay of cognitive, emotional, and social changes that accompany aging and that shape how older adults engage with the world around them (Pimentel et al., 2022). The scope of the challenge is now well documented. The World Health Organization (2022b) estimates that one in seven older adults lives with a mental disorder, with depression and anxiety especially common, and especially common among women. Older adults also account for 27% of global suicides, and one in six experiences violence, frequently from caregivers, which compounds psychological suffering. These figures exclude dementia, which affects roughly 6.9% of older people and remains a major public health concern (World Health Organization, 2022a).

This burden will intensify as populations age. The number of people aged 65 and over is projected to reach 1.5 billion by 2050, about 16% of the global population, with especially rapid growth in Latin America and in Brazil specifically (United Nations, 2024). Yet a basic asymmetry persists in the evidence base. Most of what is known about the mental health of older adults derives from high-income, predominantly WEIRD societies (Western, Educated, Industrialized, Rich, and Democratic), while the populations whose later lives unfold under entrenched material deprivation remain markedly underrepresented in psychological science (Henrich et al., 2010; Thalmayer et al., 2021). Closing this gap is more than a matter of geographic balance. Adverse conditions such as food insecurity, social isolation, and chronic illness amplify psychological suffering and erode autonomy and functional capacity (Frangos et al., 2023; Srivastava et al., 2021), and these conditions are systematically more prevalent precisely in the regions least represented in the literature.

Psychological distress provides a useful lens for this inquiry. It can be understood as the subjective manifestation of stress processes, an adaptive response to circumstances appraised as threatening or overwhelming that engages cognitive, emotional, and behavioral processes with consequences for physical and mental health (Lazarus & Folkman, 1984). In the Transactional Theory of Stress and Coping, stress arises when perceived demands exceed perceived resources. Two appraisals guide the process. A primary appraisal asks whether the situation poses a threat or a challenge, while a secondary appraisal asks whether the person has the resources needed to cope (Lazarus & Folkman, 1984). These appraisals shape subsequent responses, which may be adaptive, such as seeking social support, or maladaptive, such as withdrawal or self-destructive behavior (Wang & Suntai, 2021). Crucially, both appraisals are conditioned by the material and social circumstances in which a person lives. Distress is therefore as much a sociological as a psychological phenomenon.

Among older adults, distress often presents as anxiety, sleep problems, impaired concentration, and irritability, and each of these undermines daily functioning and independence (Wang & Suntai, 2021). Prevalence is markedly higher in contexts of vulnerability, including low income, limited schooling, multimorbidity, social isolation, and food insecurity (Aciole & Silva, 2021; Gyasi et al., 2024; Srivastava et al., 2021). Rooted in structural inequities, these conditions interact to produce cumulative disadvantage across the life course, elevating distress and reinforcing feelings of sadness, hopelessness, and helplessness (Achdut, 2023). What appears at the individual level as anxiety or low mood is, from this vantage point, a body keeping score of social conditions accumulated over decades (Phelan et al., 2010).

Food insecurity is a particularly powerful determinant. Defined as inadequate and unstable physical and economic access to sufficient, safe, and nutritious food (Brasil, 2006), it is consistently associated with elevated perceived stress and greater psychological distress (Pereira et al., 2023; Pourmotabbed et al., 2020). Evidence links food insecurity to symptoms of anxiety, anguish, and despair (Gogoi & Hazarika, 2022). Older adults experiencing food insecurity report poorer mental health and lower quality of life, and they face heightened risk for adverse health outcomes (Silva et al., 2022). Beyond its nutritional consequences, food insecurity functions as a chronic psychosocial stressor. It signals scarcity, threatens autonomy over basic decisions, and forces older adults to confront the precariousness of their material lives on a daily basis.

Findings from Northeast Brazil illustrate this pattern with particular clarity. In households with older adults served by the Family Health Strategy (Estratégia Saúde da Família, ESF), depressive symptoms have been strongly associated with food insecurity. Mild food insecurity was linked to a threefold increase in the likelihood of depressive symptoms, and moderate to severe food insecurity to a fivefold increase, relative to food-secure households (Pereira et al., 2023). International research likewise shows that food insecurity predicts depressive symptoms, anxiety, poor sleep, loneliness, and cognitive decline (Myers, 2020; Pengpid & Peltzer, 2024; Smith et al., 2021). Yet much of this evidence overlooks the specific sociocultural and economic contexts in which distinct populations age. The Brazilian Northeast is itself such a context. The region concentrates some of the country’s lowest Human Development Index scores and highest rates of food insecurity, alongside a Family Health Strategy that has expanded primary care coverage but operates under chronic resource constraints. The intersection of psychological distress and food insecurity among older adults in this setting remains underexplored, despite social inequalities that likely intensify the mental health consequences of food insecurity (Mrejen et al., 2024; Pereira et al., 2023).

The relationship between food insecurity and psychological distress is therefore not only clinical. It reflects social inequities that render older people invisible and reinforce their exclusion from systems of care. Two contributions follow from situating the question in the Brazilian Northeast. First and foremost, the region offers an empirical case in which structural deprivation is the rule rather than the exception, allowing tests of associations that may be muted in more affluent samples. Second, the findings extend the geographic reach of psychological science on aging beyond its WEIRD center of gravity, where most current evidence has been generated. Alongside these risks, certain resources appear to protect mental health in later life. Regular physical activity is associated with lower distress and with better physical and emotional functioning among older adults (Ahmad et al., 2023; Wong et al., 2023). Satisfaction with living conditions and with one’s own health reflects access to the material and symbolic resources that help older people cope when demands are high (Silva et al., 2022; Srivastava et al., 2021). The present study therefore estimates the prevalence of psychological distress among older adults in three Northeastern states and tests its association with food insecurity, sociodemographic position, and health-related behaviors. We expected food insecurity, low income, and limited schooling to be associated with higher distress, and regular physical activity and greater satisfaction with living conditions and health to be associated with lower distress.

## 2. Materials and Methods

### 2.1. Study Design

This was a quantitative, cross-sectional study designed to estimate the prevalence of psychological distress and to test its associations with food insecurity, sociodemographic position, and health-related behaviors. The study is reported in line with the Strengthening the Reporting of Observational Studies in Epidemiology (STROBE) recommendations for cross-sectional studies (von Elm et al., 2008).

### 2.2. Participants

Sample size was determined a priori through power analyses appropriate to the planned tests. With α set at 0.05 (two-tailed) and statistical power at 0.80, an independent-samples *t* test with equal allocation requires 351 participants in total to detect a small-to-medium standardized mean difference of d = 0.30, and a Pearson correlation under the same constraints requires 347 participants to detect a coefficient of r = 0.15 (Cohen, 1992; Faul et al., 2007). Recruitment proceeded through non-probability convenience sampling, resulting in a final sample of 355 participants from Brazil’s Northeast region, including participants from Paraíba (*n* = 160), Piauí (*n* = 97), and Bahia (*n* = 98). These locations were selected because of their Human Development Index (HDI) levels, which rank among the eight lowest of Brazil’s 27 states. They are Piauí (HDI = 0.697), Bahia (HDI = 0.714), and Paraíba (HDI = 0.722). Because recruitment relied on convenience sampling, the resulting sample is not representative of the older population of Northeast Brazil. The prevalence reported here should therefore be read as describing this sample rather than as a population estimate for the region.

Inclusion criteria were age 60 years or older and residence in a Northeastern state. Individuals who were not present in public spaces (e.g., streets or squares) during data collection or who did not reside in the defined localities were excluded. Because recruitment took place in community settings, older adults who were institutionalized, hospitalized, homebound, or otherwise unable to be present where data were collected are likely underrepresented. These groups tend to experience poorer health and greater distress, so their underrepresentation may bias the distress estimate, a limitation we revisit when interpreting the prevalence findings.

### 2.3. Measures

**Sociodemographic questionnaire.** A structured, objective form capturing age, gender, city and state of residence, religion, religiosity, education, income, contribution to household income, occupation, ethnicity, and marital status.

**Structured health questionnaire.** Aligned with study objectives, this instrument covered alcohol and tobacco use, regular use of sleep medication, regular use of medication for mental health (anxiety and/or depression), and engagement in physical activity. Based on responses, binary grouping variables (no/yes) were created for mean comparisons. For each behavior, participants were asked whether they currently engaged in it on a regular basis, and the answer was coded as no or yes to form the grouping variables used in the mean comparisons. For physical activity, the item referred to the regular practice of physical exercise as part of a routine rather than to incidental daily movement such as walking, so a response of yes reflects a habitual exercise practice and a response of no its absence. Participants also rated satisfaction with physical and mental health on 0–10 scales.

**Brazilian Food Insecurity Scale (EBIA, short form).** We used the adapted short version for the Brazilian population (Santos et al., 2014), originally validated by Segall-Corrêa (2004). The scale has five dichotomous items (yes/no) scored 1 for each “yes.” Food insecurity is indicated by at least one affirmative response. In the present study, the scale showed good internal consistency, with Cronbach’s alpha of 0.82 and McDonald’s omega of 0.84. The confirmatory factor analysis also indicated excellent model fit, with CFI = 0.98, TLI = 0.97, RMSEA = 0.04, and SRMR = 0.03.

**Kessler Psychological Distress Scale (K10).** The K10 consists of 10 items developed by Kessler (2003) to assess symptoms of anxiety and depression over the past four weeks. Items are rated on a five-point Likert-type scale (5 = All of the time to 1 = None of the time). The scale has been validated for screening mental distress among Brazilian older adults, showing good reliability and an optimal cutoff of total score > 14, with sensitivity of 75.47% and specificity of 85.0% (Lins et al., 2021). In the present study, the K10 demonstrated excellent internal consistency, with Cronbach’s alpha of 0.91 and McDonald’s omega of 0.92. The confirmatory factor analysis further supported the adequacy of the measurement model, showing excellent fit indices: CFI = 0.97, TLI = 0.96, RMSEA = 0.05, and SRMR = 0.04.

### 2.4. Ethical Procedures and Data Collection

The study was approved by the Research Ethics Committee (CAAE: 69651023.2.0000.5187) in accordance with Resolution No. 466/12. Data were collected face to face in public spaces or at participants’ homes. All participants received information about the study objectives, confidentiality, voluntary participation, and their right to withdraw at any time, and then provided written informed consent in duplicate. Questionnaires were administered individually (average duration ≈ 15 min), either self-completed or interviewer-administered when reading assistance was needed.

### 2.5. Data Analysis

Statistical analyses were conducted in JASP. We first screened the data for missingness and examined variable distributions. Descriptive statistics (frequencies, percentages, means, ranges, and standard deviations) were computed, followed by independent-samples *t* tests and Pearson correlations. The extent of missing data was examined for all study variables before the main analyses. There were no missing values on any of the study variables, so all 355 participants entered every analysis and no imputation or case deletion was required. The assumptions of the independent-samples *t* tests were then examined. Normality of K10 scores within each group was assessed through skewness and kurtosis values together with visual inspection of histograms and Q-Q plots, and homogeneity of variance was tested with Levene’s test. When the assumption of equal variances was not supported, the Welch correction was applied, and with 355 participants, the comparisons were also robust to moderate departures from normality. Correlations with food insecurity used the five-item EBIA total score, and the point-biserial correlation computed with the dichotomous food insecurity indicator, which corresponds to a Pearson correlation between a binary and a continuous variable, was inspected as a robustness check, together with Spearman coefficients for the satisfaction variables measured on 0 to 10 scales. To assess whether food insecurity remained associated with psychological distress once other risk factors were taken into account, a multivariable linear regression was estimated with the K10 total score as the outcome and food insecurity, age, gender, monthly income, education, regular use of sleep medication, regular use of mental health medication, physical activity, and satisfaction with physical health as predictors. The assumptions of the regression model were examined through inspection of residual versus fitted value plots for linearity and homoscedasticity, complemented by the RESET and Breusch–Pagan tests, through the distribution of the residuals, and through standardized residuals, leverage values, and Cook’s distances for influential observations. Because the residuals departed from normality and showed heteroscedasticity, the model was re-estimated with heteroscedasticity-consistent HC3 standard errors as a sensitivity analysis, and it was also refitted after excluding cases with standardized residuals above 3. The dataset used in this research program can be accessed at the Open Science Framework repository platform: https://osf.io/rbqfh/?view_only=1e35d90f7a904eea8e5d26c1c60436f7 (accessed on 24 July 2026).

## 3. Results

### 3.1. Sample Characteristics

The sample was composed primarily of cisgender women (*n* = 219; 61.7%), followed by cisgender men (37.5%) and a small proportion of transgender women (*n* = 3; 0.8%). Participants were 60–94 years old (*M_age_* = 69.00, *SD_age_* = 6.68); most (*n* = 240; 67.6%) were aged 60–69, and 32.4% (*n* = 115) were 70 or older.

Nearly all participants identified as heterosexual (99.2%; *n* = 352), and 0.8% (*n* = 3) reported another sexual orientation, with one participant identifying as homosexual, one as bisexual, and one as another orientation. Regarding education, 48.2% (*n* = 153) had up to 8 years of schooling (elementary complete or less), and 51.8% had more than 8 years. Family income was predominantly low: 63.1% (*n* = 224) reported earning up to one monthly minimum wage, while 36.9% (*n* = 131) reported higher income.

Most participants (84.8%; *n* = 301) stated that their household relied on their income; 15.2% (*n* = 54) did not have this financial responsibility. In terms of family composition, 86.2% (*n* = 306) reported having children, and 13.8% (*n* = 49) did not. Additional details appear in Table 1.

### 3.2. Psychological Distress (K10)

The mean K10 score was 18.55 (*SD* = 7.50), above the validated screening cutoff for Brazilian older adults of a total score above 14 (Lins et al., 2021). Overall, 64.5% (*n* = 229) of participants scored above this screening threshold. Food insecurity prevalence was 40.8% (*n* = 145). Because the sample was recruited by convenience and did not include older adults who were absent from public spaces during data collection, these figures are best read as describing the present sample rather than as estimates for the older population of the region. The high rate of distress should therefore be interpreted with caution.

### 3.3. Group Comparisons

Mean comparisons across sociodemographic and health variables showed no statistically significant differences in psychological distress by gender (men/women), *t*(353) = −0.713, *p* = 0.476. Distress also did not differ across the three self-identified skin color categories of White (*n* = 144, *M* = 18.10, *SD* = 6.63), Brown/Mixed-race (*n* = 134, *M* = 18.95, *SD* = 8.12), and Black (*n* = 77, *M* = 18.71, *SD* = 7.96) participants, *F*(2, 352) = 0.460, *p* = 0.632, η^2^ = 0.003. The Welch test led to the same conclusion, *F*(2, 190.4) = 0.486, *p* = 0.616, as did all pairwise comparisons and the aggregation of Black and Brown/Mixed-race participants into a single group following the IBGE classification, *t*(353) = 0.936, *p* = 0.350. Significant differences emerged for food insecurity, income, education, use of sleep medication, use of mental health medication, and physical activity. Before these comparisons were interpreted, normality and homogeneity of variance were checked for each grouping variable, and the Welch correction was applied where the assumption of equal variances was not supported. Results are presented in Table 2.

Older adults experiencing food insecurity reported significantly higher psychological distress than those without food insecurity, with a large effect size, suggesting a difference in practical relevance. Similarly, lower education and lower income were associated with significantly higher distress relative to higher education and higher income.

For health-related variables, both use of sleep medication and use of mental health medication were associated with higher distress. Notably, sleep medication use showed the largest effect size, consistent with either medication use as a response to distress symptoms or shared health demands that contribute to both outcomes.

In contrast, regular physical activity was associated with lower psychological distress, with a moderate effect size. Participants reporting no physical activity had significantly higher distress than those maintaining an exercise routine, a difference that did not persist in the adjusted model reported in Section 3.5.

### 3.4. Correlation Analyses

Pearson correlations indicated statistically significant associations between total psychological distress scores and all variables examined. Food insecurity correlated positively with distress (*r* = 0.466, *p* = 0.001), indicating higher distress with greater food insecurity. This coefficient was computed with the five-item EBIA total score, and the point-biserial correlation based on the dichotomous food insecurity indicator led to the same conclusion (*r_pb_* = 0.385, *p* < 0.001).

Satisfaction with living conditions (*r* = −0.519, *p* = 0.001), satisfaction with physical health (*r* = −0.597, *p* = 0.001), and especially satisfaction with mental health (*r* = −0.669, *p* = 0.001) were negatively associated with distress. Although all satisfaction domains related to lower distress, satisfaction with mental health showed the strongest association, underscoring its central role in psychological well-being. Spearman coefficients computed for the satisfaction variables supported the same conclusions.

**Figure 1:**
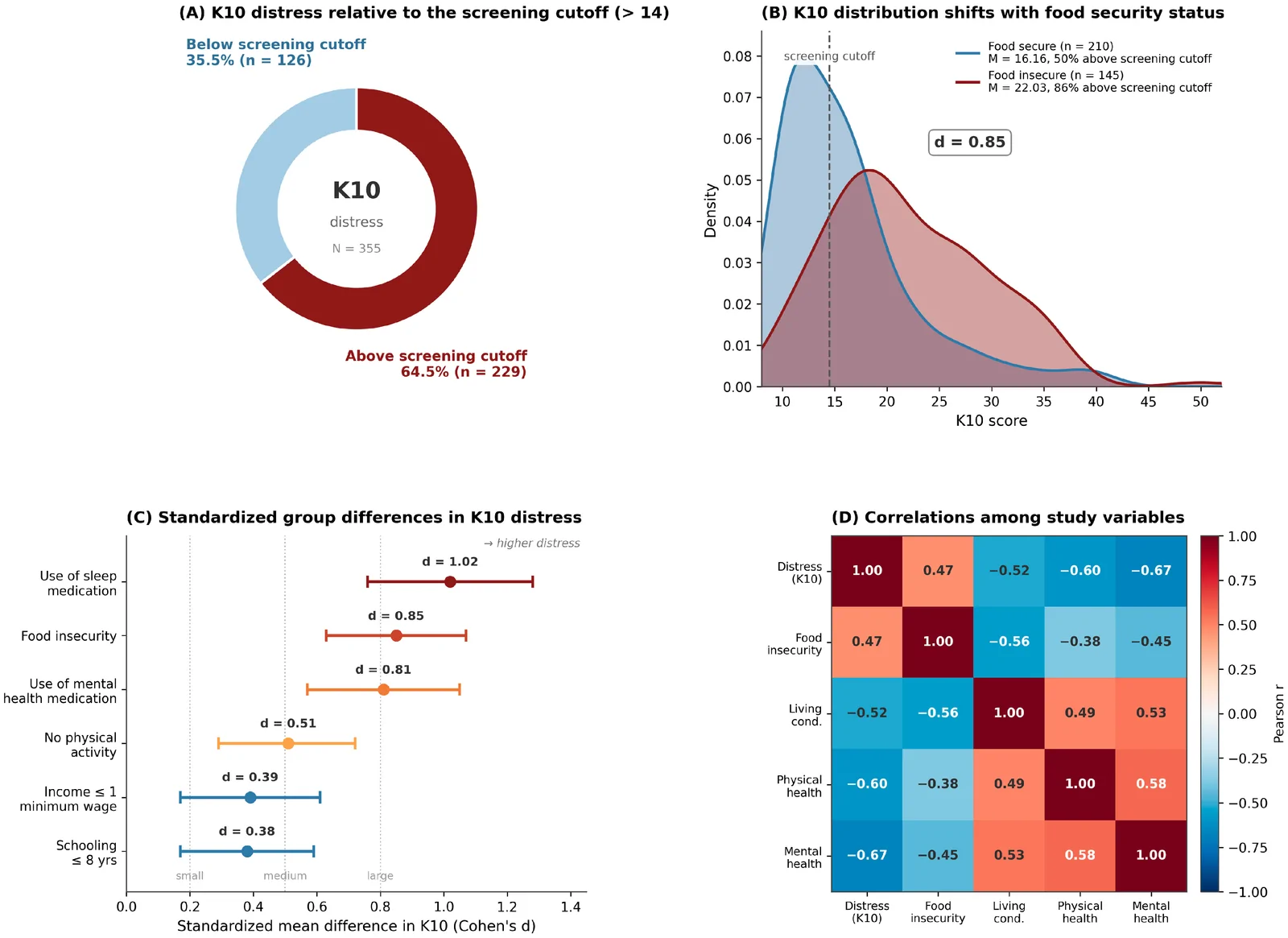
Distribution of K10 distress relative to the screening cutoff, density shift by food security status, effect sizes for risk conditions, and Pearson correlations among study variables in older adults from Northeast Brazil (*n* = 355).

Figure 1 integrates these results, presenting the prevalence of distress, the distributional shift associated with food insecurity, the effect sizes for the studied conditions, and the Pearson correlations among the continuous study variables. Panel A shows that close to two-thirds of the sample scored above the K10 screening cutoff. Panel B traces the rightward shift in K10 scores associated with food insecurity, with the food-insecure distribution sitting predominantly above the screening cutoff. Panel C orders the studied conditions by the magnitude of the standardized group difference, showing that sleep medication, food insecurity, and the use of mental health medication are associated with the largest differences in distress, while income, schooling, and physical activity show smaller but consistent differences. Panel D situates these group differences within the broader correlational structure, highlighting that satisfaction with mental health, physical health, and living conditions all relate inversely to distress, with mental health satisfaction showing the strongest single association.

### 3.5. Multivariable Analysis

To examine whether food insecurity remained associated with psychological distress once the other risk and protective factors were taken into account, the multivariable linear regression described in Section 2.5 was estimated with the K10 total score as the outcome. The model accounted for a substantial share of the variance in distress, *R*^2^ = 0.447, adjusted *R*^2^ = 0.433, *F*(9, 345) = 30.987, *p* < 0.001. Food insecurity remained a significant predictor after adjustment, *b* = 3.194, *SE* = 0.681, 95% CI [1.854, 4.534], β = 0.210, *t*(345) = 4.688, *p* < 0.001, so food-insecure participants scored about three points higher on the K10 than food-secure participants with comparable profiles on the other predictors. The same conclusion held when the five-item EBIA total score replaced the dichotomous indicator, *b* = 0.915, 95% CI [0.548, 1.281], *p* < 0.001. Regular use of sleep medication was also independently associated with higher distress, *b* = 2.347, 95% CI [0.398, 4.296], *p* = 0.018, and satisfaction with physical health was the strongest predictor in the model, with higher satisfaction relating to lower distress, *b* = −1.306, 95% CI [−1.577, −1.036], β = −0.446, *p* < 0.001. Age, gender, income, education, use of mental health medication, and physical activity did not reach significance once the other predictors were controlled, which suggests that their bivariate associations with distress overlap with the variance carried by food insecurity and perceived physical health. Multicollinearity was not a concern, with all variance inflation factors below 2. The RESET test gave no indication of nonlinearity, *F* = 0.686, *p* = 0.408, and no observation was unduly influential, with a maximum Cook’s distance of 0.086 and a maximum leverage of 0.072. The residuals were moderately right-skewed, with skewness of 0.56 and kurtosis of 1.68, and the Breusch–Pagan test indicated heteroscedasticity, LM = 37.09, *p* < 0.001, so the model was re-estimated with HC3 robust standard errors. Under robust estimation, the conclusions for food insecurity, *b* = 3.194, 95% CI [1.728, 4.660], *p* < 0.001, and for satisfaction with physical health, *p* < 0.001, were unchanged, while the coefficient for sleep medication became marginal, *p* = 0.057. Excluding the six cases with standardized residuals above 3 did not alter this pattern, and food insecurity remained a significant predictor, *b* = 3.461, *p* < 0.001. Full results appear in Table 3.

## 4. Discussion

The sociodemographic profile of our sample makes the structural backdrop visible. Most older adults lived on low incomes and with limited schooling, and many were also the primary earners in their households, all of which compound financial strain. These features mirror long-standing inequalities in Brazil’s Northeast (IBGE, 2025) and align with prior research identifying income and schooling as central determinants of social vulnerability linked to anxiety, depression, and distress in later life, particularly where social protection is fragile or insufficient (Assoumou et al., 2023; Chai & Chai, 2025; Gogoi & Hazarika, 2022; Massa & Chiavegatto Filho, 2021; Mrejen et al., 2024; Muhammad et al., 2021; Pereira et al., 2023; Pimentel et al., 2022). Read in this light, our findings should be interpreted as a portrait of psychological distress under conditions of accumulated disadvantage rather than as evidence about aging in general.

Low income is associated with chronic financial hardship, restricted access to medications and health services, and greater exposure to precarious housing conditions (Neumann et al., 2021; Pérez et al., 2022). Among older adults, these constraints are intensified by reduced work capacity and greater care needs (Gogoi & Hazarika, 2022). They limit access to adequate care, weaken support networks, and heighten exposure to stressors, increasing psychosocial vulnerability (Achdut, 2023). The fundamental cause perspective on social inequalities in health offers a parsimonious account of why these patterns recur across generations and disease categories. Resources such as money, knowledge, prestige, power, and beneficial social connections shape exposures and protections so consistently that, even as specific risks change, the social gradient in health does not (Phelan et al., 2010).

These conditions curtail access to material resources such as health care and food security, and to symbolic resources such as social support and autonomy in daily decision-making, both of which are essential for coping with the challenges of aging (Silva et al., 2022; Srivastava et al., 2021). Education, in turn, is more than years of schooling. It mediates the capacity to understand and mobilize protective strategies when facing stressors. The association of low income and low schooling with mental suffering is structural rather than incidental, reflecting inequalities accumulated across the life course (Mrejen et al., 2024; Muhammad et al., 2021; Neumann et al., 2021; Pereira et al., 2023). In the adjusted model, however, the differences by income and schooling did not remain statistically significant once food insecurity and perceived physical health were taken into account, which suggests that these positional disadvantages operate largely through material and health-related pathways rather than as independent sources of distress in this sample.

In this study, 64.5% of older adults scored above the K10 screening cutoff for psychological distress. The prevalence is at the upper end of estimates reported in older populations, which range from 26.2% to 61% (Pereira et al., 2023; Rashid et al., 2024). Differences in sampling and setting likely contribute to this elevated figure. The present sample was recruited by convenience in three states with among the lowest Human Development Index values in the country, where exposure to the conditions tied to distress is high, so comparisons with studies that used probability samples or less deprived settings should be made with caution. Two findings stand out from the correlational analyses. Lower satisfaction with living conditions was associated with higher distress, and food insecurity correlated strongly with distress, with a large effect size that is consistent with the meta-analytic estimate for this association in working-age and older samples (Pourmotabbed et al., 2020). The multivariable model reinforced this picture, since food insecurity remained associated with distress after adjustment for sociodemographic position, medication use, physical activity, and satisfaction with physical health, indicating that the association is not accounted for by the other conditions measured here.

Understanding psychological suffering in later life requires attention to living conditions (Silva et al., 2022), understood as a set of political and organizational determinants that include sanitation, housing, transportation, food, health care, education, and access to safe water (Gonçalves, 2004). In Brazil’s Unified Health System (Sistema Único de Saúde, SUS), Article 3 of the Organic Health Law No. 8.080/90 recognizes living conditions as determinants and conditioning factors of health, and assigns to the State the duty of ensuring the living conditions necessary to realize the fundamental right to health.

Evidence consistently shows that precarious living conditions predict mental illness, underscoring that mental health is inseparable from the social and material determinants of everyday life (Assoumou et al., 2023; Furtado et al., 2019). Across diverse populations, including adults, adolescents, university students, people with chronic diseases, and parents, studies in countries at varying levels of development have documented a positive association between food insecurity and psychological distress (Myers, 2020; Pourmotabbed et al., 2020). Limited and irregular access to adequate food harms physical health and functions as a chronic stressor, heightening psychological vulnerability (Gogoi & Hazarika, 2022; Osei-Owusu et al., 2024).

Mental health and well-being are, to a meaningful extent, bounded by material life conditions, which reaffirms that the guarantee of basic social rights is fundamental to mental health promotion. Material conditions operate as structural determinants that increase daily stressors, reduce coping resources, and restrict access to formal and informal support networks. Food insecurity, in particular, operates as both an acute stressor signaling immediate scarcity and a marker of chronic deprivation that potentiates psychological tension and constrains recovery. Reducing psychological suffering among older adults therefore requires intersectoral strategies that address the materiality of life and its determinants, rather than treatment paradigms that locate distress solely within the individual.

Another salient result concerns health behaviors and pharmacological use. Sleep medication showed the largest group difference in psychological distress, suggesting a tight link between mental suffering and sleep disorders in later life that has been reported in previous studies (Cuchi et al., 2024; Souza et al., 2022). In the adjusted model, this association was attenuated and became marginal under robust estimation, and satisfaction with physical health showed the largest standardized association with distress, so the bivariate prominence of sleep medication should not be read as evidence of an independent effect. Sleep quality may therefore be a particularly informative marker of mental health among adults aged 60 years and older, both as a consequence of distress and as a target for intervention. Because the design was cross-sectional, these data cannot show whether medication use precedes or follows distress. The association is best read as a marker of co-occurring sleep and mental health difficulties rather than as evidence of a causal or directional relationship.

Regular physical activity, in contrast, was associated with lower distress in the group comparisons, with a moderate effect size, echoing evidence that links exercise to lower distress, better mood, functional autonomy, and active aging (Ahmad et al., 2023; Wong et al., 2023). This association did not remain statistically significant in the adjusted model, which suggests that in this sample it overlaps with food insecurity and perceived physical health rather than operating as an independent factor. Research with long-lived older adults in São Paulo found that depressive symptoms were related to difficulties in performing basic and instrumental activities of daily living (Souza et al., 2022), reinforcing the role of physical activity for mental health and for preserving autonomy and functional capacity in contexts of social vulnerability.

In our sample, higher food insecurity was associated with lower satisfaction with physical health. Older adults who reported greater satisfaction with their physical health generally reported lower levels of food insecurity, and satisfaction with physical health was also higher among those more satisfied with their living conditions. These findings suggest that, although regular physical activity relates to better mental health, self-perceived physical health is strongly shaped by contextual factors such as living conditions and stable access to sufficient, high-quality food. They also point to the value of a positive health self-perception as a psychological resource associated with lower distress and indicate that interventions aimed at strengthening mental health may have broader effects on well-being in older age.

Pérez et al. (2022) reported, in a sample of people aged 60 and older, a concentration of poorer self-rated health among those with lower incomes. Socioeconomic factors such as income, schooling, and private health insurance contributed most to observed health inequalities, with income the principal driver. Income differences translate into disparities in other health determinants, including diet, lifestyle, and access to health services (Mrejen et al., 2024).

Although Brazil has a universal public system in the SUS, access to medical care still differs between those with and without private insurance, and lower-income individuals often face delays in obtaining necessary care (Massa & Chiavegatto Filho, 2021; Pérez et al., 2022). For older adults in the Northeast, this means that universal coverage on paper coexists with rationed access in practice, a tension that the present findings render visible.

Opportunities for health, including regular physical activity, depend on access to healthy food and dignified living conditions. Social inequities therefore weigh heavily on the older population. Material and social conditions delimit the possibilities for care, disease prevention, and health promotion, either constraining or expanding the resources available for coping. The present findings show that structural inequalities, expressed through income, schooling, and living conditions, shape psychological suffering among older adults in Brazil’s Northeast. This suffering is not reducible to individual experience. It reflects a social context marked by inequality and demands explanation at the level of social structure as much as at the level of the person. Promoting mental health in this age group requires intersectoral actions that integrate food security, universal access to health care, encouragement of physical activity, stronger support networks, and strategies to counter the stigma that links aging, poverty, and mental disorders. The implication for psychological science is equally pointed. Theories of stress, coping, and aging that have been built largely from WEIRD samples need to be tested, refined, and at times rebuilt in settings where deprivation is the dominant feature of older adults’ daily lives.

The patterning of distress also raises questions about social position that the present design could only partly address. Mean distress did not differ significantly by gender or by self-identified skin color, which sits at odds with research reporting higher distress among women and among racially marginalized older adults (Mrejen et al., 2024; Muhammad et al., 2021). Several features of this study may account for the absence of such differences. The sample was recruited by convenience from settings where deprivation is widespread, which may compress the variation that would otherwise track gender or skin color. Distress did not differ across the three self-identified skin color categories, and gender included categories with small subgroups, so the power to detect differences within specific subgroups and the capacity to examine how these markers combine remain limited. Because gender, skin color, income, and schooling are likely to shape mental health jointly rather than in isolation, their separate effects can be muted when each is considered on its own. Research in this region would benefit from measures that capture social position in finer detail and from analyses designed to model the joint influence of these markers.

## 5. Conclusions

This study examined the prevalence of psychological distress among older adults in Brazil’s Northeast and its association with food insecurity, sociodemographic position, and selected health-related behaviors. Distress was common in this sample and occurred alongside material hardship. Older adults with food insecurity, lower schooling, lower income, the use of psychotropic or sleep medication, and no regular physical activity reported higher distress in the group comparisons, and food insecurity remained associated with distress after adjustment for sociodemographic and health-related covariates. Because the design was cross-sectional and the sample was recruited by convenience, these patterns describe associations within a vulnerable group rather than causal relationships or estimates for the older population as a whole, and the reliance on self-reported screening measures and the confounders that were not comprehensively modeled call for the same caution.

The implications follow naturally. Advancing the mental health of aging populations in low-resource settings requires policy that is as intersectoral as the problem itself, joining income support and food security with accessible community-based mental health care, routine screening for distress and food insecurity in primary care, and environments that support physical activity and social connection. Future work should move from description toward explanation and evaluation, with longitudinal and multilevel designs that clarify temporal ordering and separate individual from contextual effects, and with intervention research embedded in primary care that tests whether measures such as food support and brief psychosocial care reduce distress in this population.

These findings point to the practical value of addressing food security and access to care alongside mental health support for older adults living under structural deprivation. They also illustrate what mental health research can show when it is carried out in a non-WEIRD setting where scarcity is a routine feature of later life, since frameworks developed mainly in affluent samples gain reach and accuracy when they are tested under such conditions.

## Figures and Tables

**Table 1:** Sociodemographic characteristics of the participants (*n* = 355).

Variables	Categories	Frequency (%)
Education Background	Incomplete elementary/middle school	95 (26.8%)
	Completed elementary/middle school	58 (16.3%)
	Incomplete high school	18 (5.1%)
	Completed high school	92 (26.0%)
	Higher education	57 (16.1%)
	No formal education	35 (9.9%)
Labor Status	Retired	247 (69.6%)
	Formal employment	49 (13.8%)
	Informal employment	40 (11.3%)
	Unemployed	19 (5.4%)
Skin Color	White	144 (40.6%)
	Brown/Mixed-race	134 (37.7%)
	Black	77 (21.7%)
Marital Status	Married	161 (45.4%)
	Living with a partner	55 (15.5%)
	Single	58 (16.3%)
	Separated, divorced, or widowed	81 (22.8%)
Religion	Catholic	180 (50.7%)
	Evangelical	120 (33.8%)
	African-matrix religion	20 (5.6%)
	Others	35 (9.9%)

**Table 2:** Comparison of mean psychological distress scores between criterion groups (*n* = 355).

Variables/Compared Groups	*n*	*M* (*SD*)	*t*	*p*	*d*
Food Security Status					
*Food Secure*	210	16.16 (6.52)	−7.847	0.001	−0.847
*Food Insecure*	145	22.03 (7.48)			
Educational Background					
*Up to 8 years of education*	188	19.86 (8.38)	3.540	0.001	0.376
*More than 8 years of education*	167	17.08 (6.05)			
Income					
*≤1 minimum wage*	224	19.61 (8.29)	3.528	0.001	0.388
*>1 minimum wage*	131	16.75 (5.48)			
Use of Mental Health Medication					
*No*	259	17.01 (6.64)	−6.780	0.001	−0.810
*Yes*	96	22.73 (8.10)			
Use of Medication to Sleep					
*No*	276	16.99 (6.51)	−8.001	0.001	−1.021
*Yes*	79	24.04 (8.17)			
Regular Physical Activity					
*No*	140	20.79 (8.34)	4.654	0.001	0.505
*Yes*	215	17.10 (6.51)			

*Note. t* = *t*-test; *M* = Mean; *SD* = Standard Deviation; *d* = Cohen’s *d*; *p* < 0.05. The *t* and *d* values are signed as the first listed group minus the second listed group in each comparison. Food security status was assessed with the short-form Brazilian Food Insecurity Scale, where food secure indicates no affirmative responses and food insecure indicates at least one affirmative response. Regular physical activity records whether participants reported a regular exercise routine rather than incidental daily movement such as walking, coded as no or yes. Educational background was grouped as up to eight years or more than eight years of formal schooling.

**Table 3:** Multivariable linear regression predicting psychological distress (K10) (*n* = 355).

Predictor	*b*	*SE*	95% CI	β	*t*	*p*
Food insecurity (1 = food insecure)	3.194	0.681	[1.854, 4.534]	0.210	4.688	<0.001
Age (years)	−0.052	0.046	[−0.144, 0.039]	−0.047	−1.132	0.258
Gender (1 = woman)	−0.348	0.648	[−1.622, 0.926]	−0.022	−0.537	0.592
Monthly income (1 = more than one minimum wage)	−0.580	0.731	[−2.018, 0.858]	−0.037	−0.794	0.428
Educational background (1 = more than eight years)	0.212	0.697	[−1.159, 1.584]	0.014	0.305	0.761
Use of medication to sleep (1 = yes)	2.347	0.991	[0.398, 4.296]	0.130	2.368	0.018
Use of mental health medication (1 = yes)	1.360	0.899	[−0.409, 3.129]	0.081	1.512	0.131
Regular physical activity (1 = yes)	−0.883	0.652	[−2.165, 0.398]	−0.058	−1.356	0.176
Satisfaction with physical health (0 to 10)	−1.306	0.138	[−1.577, −1.036]	−0.446	−9.498	<0.001

*Note. b* = unstandardized regression coefficient; *SE* = standard error; CI = confidence interval; β = standardized coefficient. Model fit, *R*^2^ = 0.447, adjusted *R*^2^ = 0.433, *F*(9, 345) = 30.987, *p* < 0.001. Binary predictors were coded 0/1, with the category coded 1 shown in parentheses. All variance inflation factors were below 2.

## Data Availability

The data from this study are available on the Open Science Framework platform: https://osf.io/rbqfh/?view_only=1e35d90f7a904eea8e5d26c1c60436f7 (accessed on 24 July 2026).
